# Mind over mood: exploring the executive function’s role in downregulation

**DOI:** 10.3389/fpsyg.2024.1322055

**Published:** 2024-01-25

**Authors:** Jose A. Rodas, Jose Leon-Rojas, Brendan Rooney

**Affiliations:** ^1^Escuela de Psicología, Universidad Espíritu Santo, Samborondón, Ecuador; ^2^School of Psychology, University College Dublin, Dublin, Ireland; ^3^Escuela de Medicina, Universidad de las Américas, Quito, Ecuador

**Keywords:** executive function, emotional downregulation, cognitive flexibility, multitasking, switching, working memory, inhibitory control

## Abstract

Emotion regulation plays a key role in well adapted behaviour, however, factors influencing individual differences in ER are still under investigation. Across two studies we investigate the complex relationship between executive functions (EFs) and emotional downregulation through two complementary research designs. The focus lies on key components of EFs—working memory, inhibitory control, and switching—and their relationship with effective emotional regulation. Surprisingly, switching emerged as the sole significant predictor in two multiple linear regression models, challenging the conventional belief that all major EFs broadly contribute to emotional downregulation. The first study, involving 248 Ecuadorian adults between 18 and 60 years old, used experimental tasks to assess the association between EFs and emotional regulation, aligning with existing literature that posits a link between EFs and emotional control. The second study, involving 180 Ecuadorian adults between 18 and 43 years old, added depth by incorporating self-report measures, providing a broader, ecologically valid perspective. However, these measures did not significantly predict downregulation, highlighting a gap between self-perception and actual cognitive abilities. Additionally, demographic predictors varied between the two studies, urging future research to consider methodological design and task selection carefully. The study also raises questions about the validity of commonly used measures, emphasising the need for more nuanced tools to capture the complexity of EFs and emotional regulation. Our findings suggest a targeted research avenue focusing on EFs for both future research and clinical interventions. Attention is called to the methodological decisions that can influence the observed associations, and the need for broader demographic representation in future studies.

## Introduction

Emotion regulation is a critical aspect of human functioning that allows individuals to manage and adjust their emotional responses to various situations. Effective regulation of emotions has been linked to a range of positive outcomes, such as improved social functioning, psychological well-being, and physical health (Gross and John, 2003; Ford et al., 2017; Chervonsky and Hunt, 2019). However, these same studies highlight the role of individual differences in regulation of emotions, with some individuals displaying greater ability to regulate their emotions than others.

### Up- and downregulation of emotions

This process encompasses a spectrum of strategies that individuals deploy to modulate their emotional responses to various situations. These strategies may aim either to intensify the emotional experience and expression—referred to as up-regulation—or to decrease their intensity, known as downregulation. Up-regulation of emotions involves cognitive and behavioural approaches designed to amplify or sustain a particular emotional state. The primary objective is often to augment positive emotions, thus enhancing overall well-being. Empirical research suggests that up-regulation can be effectively achieved through a variety of cognitive reappraisal techniques, such as accentuating the positive aspects of a situation or elevating its personal significance (Kim and Hamann, 2007; Gyurak et al., 2012).

Conversely, downregulation of emotions entails cognitive and behavioural methods focused on reducing or moderating the intensity of specific emotional states. The primary aim is generally to mitigate negative emotions or to promote adaptive functioning in emotionally challenging contexts. Common techniques for achieving this include cognitive reappraisal methods, such as reinterpreting a situation in a less emotionally charged manner (Gross, 1998). Functional magnetic resonance imaging (fMRI) studies (Ochsner and Gross, 2005; Goldin et al., 2008) have shown that when individuals effectively down-regulate emotions, there is increased activation in brain regions associated with cognitive control and emotional processing.

Empirical studies provide robust support for the efficacy of downregulation strategies. For example, a meta-analysis by Webb et al. (2012) highlighted that downregulation can lead to favourable outcomes in both emotional experiences and behaviours. Likewise, research by Aldao et al. (2010) showed that successful downregulation correlates with reduced psychological stress and fewer symptoms of emotional disorders like anxiety and depression. Effective downregulation is instrumental in maintaining both social and emotional well-being (Deng et al., 2013; Zou et al., 2019). Specifically, individuals who effectively manage to down-regulate their emotions tend to exhibit better behavioural control in social contexts, sustain more positive relationships, and cope more adeptly with stress (Gross, 2002; Monteiro et al., 2014).

Numerous studies have investigated individual differences in downregulation of emotions, and these studies have identified several factors that contribute to these differences, such as impulsivity and adjustment (Zou et al., 2019) or degree of activation of different brain regions for up-and downregulation (i.e., emotional experience regions for up-regulation and regions receiving interoceptive input for downregulation; Min et al., 2022). One set of key factors expected to influence individual differences in downregulation of emotions are the executive functions (EFs).

### Executive functions and downregulation

EFs are a set of cognitive functions specifically aimed at orchestrating other cognitive processes to significant goals (Baddeley, 1996; Diamond, 2013). In other words, these set of functions allow humans to direct thought and behaviour to significant goals. From this perspective, EFs and downregulation should be closely intertwined, as both are predominantly governed by the prefrontal cortex in the brain (Ochsner and Gross, 2005; Ochsner et al., 2009), and serve the purpose of controlling and directing behaviour in a specific direction. The prefrontal cortex is responsible for higher-order cognitive processes, such as planning, decision-making, and problem-solving.

In situations requiring emotional control, the prefrontal cortex exerts an inhibitory influence on the limbic system (Ochsner and Gross, 2005; Moodie et al., 2020), a primitive part of the brain involved in generating emotional responses. By doing so, the prefrontal cortex would help to modulate emotional reactions, making them more nuanced and context-appropriate. For example, when a person is feeling angry or frustrated, the executive functions would allow to evaluate the situation rationally, consider the consequences of acting on emotional impulses, and then choose a more constructive course of action. Despite the conceptual and neural overlap between EFs and emotion regulation, the two constructs have been mostly studied as separate entities.

Research has identified three distinct executive functions (Miyake et al., 2000; Diamond, 2013): Working memory, as an individual’s ability to hold and manipulate information in mind; multitasking or switching, as the ability to switch the focus of cognitive resources between mental sets; and inhibitory control, as the ability to inhibit preponderant responses when considered inappropriate. Studies have shown that individuals with higher working memory capacity are better able to down-regulate their emotions (Schmeichel et al., 2008; Schmeichel and Demaree, 2010; Hendricks and Buchanan, 2016), possibly because they are better able to focus their attention or deploy more cognitive resources on regulating their emotional responses. In contrast, individuals with lower working memory capacity may be less able to sustain attention on regulating their emotional responses, leading to less effective downregulation of emotions.

Research exploring the role of inhibitory control in emotional regulation has yielded surprisingly inconclusive results (e.g., McRae et al., 2012; Beauchamp et al., 2016; Hendricks and Buchanan, 2016; Sperduti et al., 2017). For example, a study by Gärtner et al. (2022) conducted an exhaustive assessment of inhibitory control, generating a latent score based on six different experimental tasks focused on this cognitive function. Concurrently, emotion regulation was evaluated through a combination of self-report instruments and psychophysiological markers. Despite employing a robust methodological approach and contrary to prevailing expectations, the study found no significant relationship between these two variables, which are conceptually related. This unexpected finding raises questions about the underlying mechanisms connecting inhibitory control and emotional regulation, warranting further investigation.

Interestingly, despite the anticipated role of switching in the downregulation of emotions there is a lack of evidence to support its participation in ER. One would reasonably expect that switching capabilities would facilitate reappraisal, refocusing, or the putting into perspective of emotional experiences. Such cognitive processes could also serve as protective factors against the employment of strategies like catastrophising or rumination, widely understood as dysfunctional (Ford et al., 2017; Dryman and Heimberg, 2018). This absence of empirical support is in part attributable to a scarcity of research on the subject, with the studies available, as in the case of inhibition, providing inconsistent results (e.g., McRae et al., 2012; Whitmer and Gotlib, 2012; Malooly et al., 2013; Hendricks and Buchanan, 2016; Liang et al., 2017; Sperduti et al., 2017).

The inconsistent evidence regarding the influence of specific executive functions on emotion regulation suggests that the relationship between these two domains is likely nuanced and may operate through particular pathways that remain to be fully elucidated. Conceptually and neurologically, one would anticipate a stronger association between cognitive and emotional control mechanisms than current studies reflect. For example, inhibitory control is by definition expected to play a vital role in emotion regulation, given its function in suppressing prepotent responses, which should extend to emotional impulses. Likewise, working memory could be implicated in emotion regulation by holding emotional information temporarily and modifying it for appropriate emotional responses. The ability to switch attention or multitask, known as cognitive flexibility, is also thought to be essential in emotion regulation, as it allows individuals to shift their focus away from emotionally distressing stimuli or thoughts, thereby facilitating more adaptive emotional responses.

A significant contribution to the study of the relationship between EFs and ER is provided by Pruessner et al. (2020). In their article, they consolidate empirical evidence concerning this relationship and propose that inconsistencies in findings may be attributed to the differing demands on EFs exerted by various ER tasks, particularly when cognitive control requirements are high. Their framework primarily focuses on emotion regulation flexibility under varying contextual demands. This involves the individual’s capacity to (a) switch or discontinue certain strategies, (b) maintain strategies, and (c) monitor the situation and the deployment of these strategies.

From a neurological standpoint, both executive functions and emotion regulation share underlying neural circuits, particularly in regions such as the prefrontal cortex and the limbic system (Ochsner and Gross, 2005; Moodie et al., 2020). The prefrontal cortex, known for its role in complex cognitive processes, also interacts with the amygdala, a key structure in emotion processing, to modulate emotional responses. This neurobiological interconnectivity further supports the notion that executive functions and emotion regulation are inherently linked and work in concert to contribute to adaptive human behaviour.

The biological underpinnings of differences in emotion regulation and executive functions require an exploration of demographic factors that may influence these effects, such as sex and age. Studies suggest a general improvement in emotion regulation with age. Masumoto et al. (2016), for instance, observed enhanced emotion regulation in men with increasing age, highlighting the combined effects of age and sex. Similarly, McRae et al. (2008) investigated emotion regulation in response to emotionally charged images. They found that men exhibited a greater reduction in amygdala activity and lower activation in prefrontal regions associated with EFs and ER, including the anterior cingulate and both superior and inferior frontal gyri, compared to women. While the specific roles of sex and age in emotion regulation are yet to be definitively established, their impact is clearly significant and must be considered in such research.

### The current study

The present research aims to further explore the relationship between EFs and emotional downregulation. Specifically, the study seeks to determine whether higher cognitive abilities in working memory, inhibitory control, and switching—key components of EFs—are predictive of effective emotional downregulation. Given the exploratory nature of the current study, we do not have specific hypotheses to test. Employing an integrative approach that combines both experimental tasks and self-report measures, this research explores to what extent individual differences in EFs predict emotional regulation capabilities across two studies. In the first study, we place an emphasis on examining the relationship between working memory, inhibitory control, and switching with the capacity for emotional downregulation.

For the second study, our aim was to verify whether the findings from the first study could be replicated and to examine more thoroughly the relationship between EFs and ER. Consequently, this study builds upon these initial findings by incorporating self-report measures, thus offering a more comprehensive and ecologically valid perspective on the role of executive functions in day-to-day emotional regulation. Although both studies focus on evaluating the same dimensions of executive functions, they employ different cognitive tasks for this assessment, thereby enhancing the robustness and generalisability of our findings. Emotional regulation in each study is assessed using the same task, requiring participants to self-report the intensity of their emotions after being exposed to images designed to evoke high levels of emotional arousal.

## Method

### Study 1

#### Participants

The sample consisted of 248 healthy adults between 18 and 60 years old (*M* = 22.73, SD = 6.36, 169 female). Participants were adults recruited from the school of psychology from Universidad de Guayaquil in Guayaquil and Universidad de las Américas in Quito, both from Ecuador. Participants were asked to share the study with their friends and relatives.

#### Instruments

The assessment in Study 1 involved a brief demographic questionnaire (age, sex, marital status and level of education) and a set of experimental tasks meant to evaluate executive functions and emotion regulation. In many EF tasks, the differences in mean response times between conditions, commonly referred to as response time cost, are typically employed as outcome scores. However, the reliability of these measures is often questionable, as noted by Khng and Lee (2014). As a result, we opted to use error rates as our primary outcome measure, due to their greater reliability, as evidenced in studies by Hedge et al. (2018) and Hughes et al. (2014).

##### Colour word Stroop task

**Figure 1:**
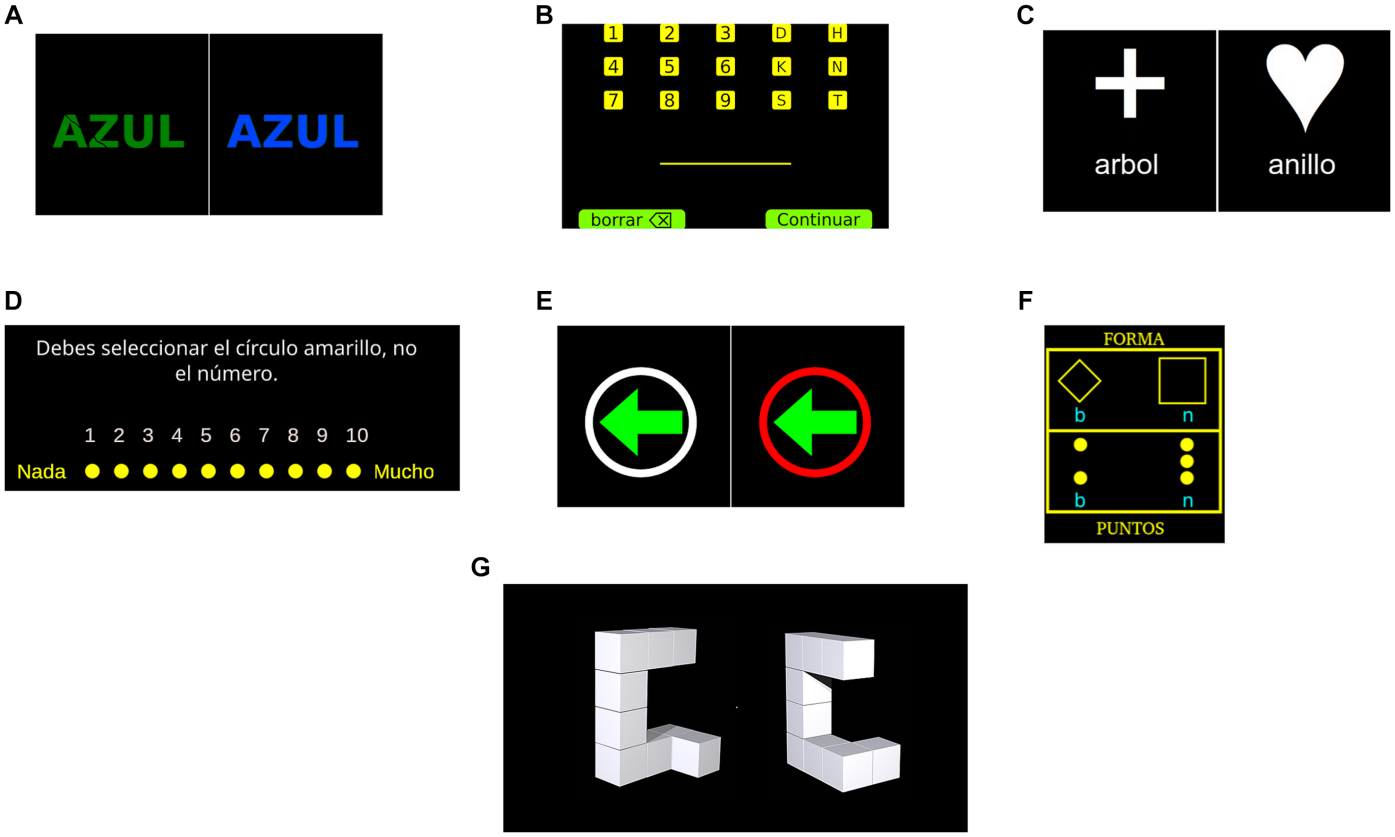
Graphical depiction of experimental tasks. **(A)** Features two distinct stimuli: ‘azul,’ which translates to ‘blue’ in Spanish, is displayed on the left as an incongruent stimulus, while a congruent stimulus is presented on the right. **(B)** Shows the keyboard layout provided to participants, featuring the options ‘borrar’ (delete) and ‘continuar’ (continue) at the bottom of the screen. **(C)** Displays two different stimuli: ‘arbol,’ which is ‘tree’ in Spanish, and ‘anillo,’ meaning ‘ring.’ **(D)** Illustrates an example of the rating scale that is presented to participants after each picture, complete with instructions in Spanish. **(E)** Portrays two different stimuli: on the left, an arrow without the ‘nogo’ indication, and on the right, a red circle signifying a ‘nogo’ trial. **(F)** Contains the words ‘FORMA’ and ‘PUNTOS,’ which translate to ‘shape’ and ‘dots’ in Spanish, respectively. Finally, panel **(G)** shows figures that are not identical.

A computerised version of the Stroop task (Stroop, 1935; MacLeod, 1991) was used in the study, which is a common measure of inhibitory control in experimental settings. In this version of the task, participants were presented a word over a black screen for which a response must be given in the form of a keypress. The word could be either *rojo*, *verde* or *azul* (red, green and blue in English, respectively) printed in either red, green or blue coloured text. Participants were asked to respond according to the colour of the word and ignore the word itself and to work as fast as possible without making mistakes. This required inhibiting the automatic response of reading a word when presented. Stimuli (i.e., words) could be either congruent, when the word and colour matched (right side of Figure 1A), or incongruent, when these did not match (left side of Figure 1A). Participants first completed a set of practice trials, requiring 10 correct responses to proceed to the evaluation phase of the task. During this block of practice trials, participants received feedback (i.e., ‘correct’ or ‘incorrect’) after each response. The evaluation phase consisted of 120 trials, 60 being congruent and 60 incongruent. Stimuli were presented in a pseudorandom order and the outcome variable was the error rate from the incongruent stimuli. The following procedure was used for presenting the stimuli: a black screen for 500 ms, a fixation cross for 200 ms, a black screen for 100 ms, the stimulus was presented for 5 s or until the participant pressed a key and a black screen for 500 ms.

##### Letter-digit sequencing task

The Letter-digit sequencing task (Crowe, 2000) is commonly used for evaluating working memory, since it requires participants to remember and manipulate information in short-term memory. In this task, participants were presented with sets of characters that could be digits from 1 to 9 or the letters ‘D’, ‘H’, ‘K’, ‘N’, ‘S’ or ‘T.’ These characters would be randomly combined to form sets of different lengths, ranging 3 to 9 characters per set. Each character from a set would be presented one at a time over a black screen for 800 ms with an interstimulus interval of 200 ms. After presenting all characters from the set a digital keyboard was shown allowing for the participant to introduce their response by clicking at each key (see Figure 1B). The keyboard also allowed participants to delete characters when needed. Participants were asked to introduce all digits first in ascending order and then the letters in alphabetical order. Two sets were created for each possible length of characters (e.g., two sets of three characters, two sets of four characters, etc.). Participants were first presented with three-character sets, if at least one of these sets was recalled correctly a longer set was presented. This procedure was repeated until the participant failed to recall at least one set of a given length or the maximum length of 9 characters was achieved. The total number of correctly recalled sets was used as the outcome variable.

##### Dual task 1

This paradigm has been used in previous studies (Miyake et al., 2000) to assess switching proficiency, specifically the ability to fluidly transition attention between different cognitive sets. Participants engaged in a classification task wherein they categorised words based on either the comparative size of the objects they represented (larger or smaller than a standard football), or by determining if the words represented a living entity. In each trial, a word and a symbolic cue were displayed at the centre of a black screen. The symbolic cue directed the categorisation strategy for the word. A cross symbol indicated a size-based classification (left side of Figure 1C), while a heart symbol denoted a living/non-living classification (right side of Figure 1C). These word-symbol pairings were pseudorandomly generated, resulting in two key task conditions: a no-switch condition and a switch condition. The no-switch condition required the same categorisation method as the preceding trial, while the switch condition necessitated a different categorisation strategy. The stimuli remained on-screen until the participant provided a response via a keypress, followed by an intertrial interval of 1 s. To ensure familiarisation with the task, participants first completed three sets of practice trials, each requiring a minimum of five correct responses. The first set required classification based on object size, the second set involved classification based on living status, and the final set incorporated a pseudorandom mix of both categorisation types. Following these practice sets, participants responded to a set of 52 trials. Both response time and error rate for the switch and no-switch conditions were recorded, but only error rate in switch trials was utilised as the primary outcome metric.

##### Emotional experience task

To assess emotional experience, we employed a widely used methodology in the study of emotion regulation (Ochsner et al., 2004; McRae et al., 2008; see Ochsner et al., 2012 for a review). This involved presenting participants with emotionally evocative pictures and then asking them to rate their experiences. Participants were instructed either to regulate or not their emotional responses. Previous studies using this method have noted differences in the amygdala and prefrontal brain regions associated with emotion and emotion regulation. These differences were particularly evident when participants were asked to regulate their emotions in response to pictures of high emotional valence.

In the current version of the task, participants were exposed to a total of 70 images sourced from the International Affective Picture System (IAPS; Bradley and Lang, 2017). The list of pictures used can be found in Supplementary Table S5. Of these, 50 were categorised as highly aversive, while the remaining 20 were deemed emotionally neutral. Following the immediate display of each image, participants were prompted to quantify the intensity of their emotional experience. The study encompassed two distinct conditions, each comprising 35 images. During the initial condition, participants were simply directed to observe the images and subsequently quantify their emotional state, a mode referred to as ‘free experience.’ Conversely, in the second condition, participants were explicitly instructed to control their emotions in order to experience them less intensively. For instance, they were advised to think of the pictures as unreal or to use any other strategy they found helpful. This guidance was intended to help participants actively manage their emotional responses. We termed this condition as ‘controlled experience.’ Each experimental trial commenced with a 4-s instruction, directing participants to either regulate their emotions (‘controlled experience’) or to refrain from doing so (‘free experience’). This was succeeded by a 6-s presentation of the stimulus image. Participants were then presented with a 10-point rating scale, allowing them to indicate the magnitude of their emotional experience (Figure 1D). The resultant outcome score was computed as the difference between the mean scores in the ‘free experience’ and ‘controlled experience’ conditions, with higher numerical values serving as indicators of superior emotional regulatory capacity. Although the condition and pictures were presented in a random order, this order was fixed for all participants.

### Study 2

#### Participants

In this study, 180 participants between 18 and 43 years old (*M* = 22.14, SD = 4.34, 123 female) were recruited from the same locations as in Study 1, Universidad de Guayaquil and Universidad de las Américas, both universities from Ecuador. Participants were incentivised to share the study with friends and family.

#### Instruments

In Study 2, the same demographic questionnaire from Study 1 was used (age, sex, marital status and level of education), as well as a set of experimental tasks and two questionnaires evaluating executive functioning. As in Study 1, error rates were preferred over response time costs due to their higher reliability as outcome scores in cognitive tasks.

##### The stop-signal task

The stop-signal task (SST; Logan, 1994) was utilised to evaluate the participants’ inhibitory control. During this task, participants were directed to respond both promptly and accurately to a visual ‘go’ stimulus, represented by a left-or right-pointing arrow enclosed in a white circular frame (refer to Figure 1E, left side). This ‘go’ signal was presented on-screen during every trial. Participants were to use the ‘A’ and ‘L’ keys to indicate the direction of the arrow for the left and right directions, respectively. In approximately 33% of the trials, a ‘stop’ signal – characterised by the white circular frame transitioning to red – succeeded the ‘go’ stimulus at a variable delay (refer to Figure 1E, right side). Upon the presentation of the ‘stop’ signal, participants were expected to refrain from responding to the ‘go’ stimulus, constituting a no-go trial. The initial stop-signal delay (SSD) was predetermined at 250 ms and was adjusted as per a staircase procedure. Successful inhibition of the response in a stop trial led to an increment of 50 ms in the SSD for the next stop trial, thereby increasing the task’s difficulty. If the participant failed to inhibit their response, the SSD was decreased by 50 ms, simplifying the task. This method ensured that participants maintained an approximate inhibition rate of 50%, enabling the computation of the stop-signal reaction time (SSRT). The SSRT was determined by subtracting the mean SSD from the average reaction time of the go trials. This offered an estimate of the time needed for participants to withhold a response post the presentation of the stop-signal. Each trial began with a fixation cross displayed for 500 ms, followed by the stimulus (an arrow within a circle), which was presented for 750 ms or until a key was pressed. An intertrial interval of 500 ms was maintained. Prior to the main task, participants initially completed a set of practice trials comprising solely go trials. A minimum of 20 correct responses were required for successful completion of this set. Subsequently, they engaged in another practice set containing 15 go trials and 10 no-go trials, randomly sequenced. In both scenarios, immediate feedback was provided after each trial along with the mean response time at the end of the set. Following these practice runs, participants proceeded to undertake two blocks of 105 trials each, with 70 being go trials.

##### Dual task 2

Similar to Dual Task 1, participants were asked to execute two alternating classification tasks. For illustrative purposes, both tasks are presented simultaneously in Figure 1F. They were presented with a visual stimulus, either a square or a diamond, which contained two or three dots. The task required participants to press a key corresponding either to the number of dots or to the shape, with the specific classification depending on the figure’s position on the screen. If the stimulus was displayed in the upper region of the screen, participants were instructed to respond based on the shape. Conversely, if the stimulus was shown in the lower section, the response was to be based on the number of dots. The task was divided into two conditions: a no-switch condition, which required responses to the same type of stimulus (e.g., shape-shape), and a switch condition, where participants were required to respond to both repeated and differing types of stimuli. To prepare the participants for the main task, three sets of practice trials were administered prior to the main assessment. The first set necessitated shape categorisation (no-switch), the second set required dot categorisation (no-switch), and the final set introduced both conditions (switch) in a pseudorandom order. Each of the initial two practice sets contained 10 stimuli, while the third and final practice set consisted of 20 stimuli. Upon the conclusion of the practice trials, participants then proceeded to three similar sets of trials. However, in this phase, each of the first two sets comprised 48 trials, while the third set contained 96 trials. Feedback was provided to participants whenever an error was made, both in the practice and assessment sets. The primary outcome measure was the error rate observed in the switch condition.

##### Mental rotation

The Mental Rotation task (Ganis and Kievit, 2015) was designed to assess non-verbal (visuospatial) working memory. It requires participants to store and manipulate non-verbal information, a process integral to this aspect of working memory (Hertzog and Rypma, 1991; Hyun and Luck, 2010; Lehmann et al., 2014). During each trial, participants encountered two three-dimensional figures. These figures could either be identical but oriented differently, or they could be similar yet distinct entities (refer to Figure 1G). When the figures were identical, the second was rotated along the vertical axis. Participants were instructed to determine, via a keypress, whether the figures were similar or different. The stimuli for the task were sourced from an open-access stimulus set (Ganis and Kievit, 2015). Each stimulus remained visible for a duration of 10 s or until a response was elicited, separated by an intertrial interval of 200 milliseconds. Initially, participants were presented with instructions, followed by a set of five practice trials that offered feedback on the accuracy of their responses. Subsequent to the practice trials, participants undertook a set of 96 trials. The dependent variable for this study was the error rate.

##### Emotional experience task

This task was identical to the one used in Study 1.

##### Amsterdam executive functions inventory

The Amsterdam Executive Functions Inventory (AEFI; Van Der Elst et al., 2012) is a self-report questionnaire designed to assess various aspects of executive functioning, primarily in adolescents and adults. This inventory focuses on evaluating Attention, Self-Control and Self-Monitoring, and Planning and Initiative. Unlike performance-based tests, the AEFI aims to capture how individuals perceive their own executive functioning in daily life, thus providing a more ecological perspective. The inventory comprises 13 items, each requiring a three-point rating (1 = not true; 2 = partly true; 3 = true). Higher scores signify superior executive functioning. The original English questionnaire was translated into Spanish by one of the researchers involved in this study. It was then back-translated by another researcher who is proficient in English but had no prior experience with the inventory. The back-translated version did not exhibit any significant discrepancies when compared with the original questionnaire.

##### Executive skills questionnaire-revised

The Executive Skills Questionnaire-Revised (ESQ-R; Strait et al., 2020) is a self-report inventory designed to quantitatively evaluate individuals’ executive functioning skills. This revised version contains 25 items and assesses various aspects such as plan management, time management, organisation, emotional regulation, and behavioural regulation. Participants are required to rate their experiences on a four-point Likert-type scale, ranging from 0 (never or rarely) to 3 (very often), in relation to a series of difficulties that necessitate executive skills. These difficulties include acting on impulse, losing items, or facing challenges in achieving long-term goals. Higher scores on each scale indicate difficulties in a specific skill area. The process of adapting the ESQ-R into Spanish followed a similar procedure to that employed for the AEFI. One of the researchers on this project translated the items into Spanish, and another researcher, proficient in English, subsequently back-translated them into English. No significant differences were observed between the two versions.

#### Procedure

Both studies received ethical approval by the research council from the Faculty of Psychology from Universidad de Guayaquil. The procedure was very similar for both studies, although not identical. All data was collected online using PsyToolkit (Stoet, 2010, 2017), a free and flexible tool for conducting online surveys and psychological experiments. Studies were advertised in two universities in Ecuador, Universidad de Guayaquil and Universidad de las Américas. No compensation was offered for participating and all participants were incentivised to share the link to the study. In Study 1, participants were first asked to indicate their sex, age, marital status, and level of education, followed by the Emotional Experience Task and a Spanish adaptation of the Big Five Inventory II. Data from the latter questionnaire will be reported elsewhere. The last section of the study consisted of the cognitive task presented at a random order for each participant. In Study 2, participants completed the same demographic questions, followed by the Emotional Experience Task, the AEFI, the ESQ-R, and the executive functions tasks. The latter were presented in a random order.

#### Data handling and analyses

For the analyses in both studies, descriptive statistics are provided for each of the investigated variables. Pearson’s correlation coefficients between them are presented in Supplementary materials. In each task, scores above three standard deviations from the mean were considered as outliers and removed before analyses. The predictive power of cognition over downregulation was investigated using multiple linear regression modelling following a hierarchical procedure. For both studies, the first model includes sex and age as predictors, the three subsequent models incorporate an executive function at a time according to the correlation coefficient presented with the downregulation score. In the case of Study 2, a fifth model is analysed including three scores from the questionnaires evaluating executive functions. The first of these scores corresponds to the attention sub-scale from the AEFI, the second and third are composite scores built from averaging the scores from several sub-scales from the AEFI and ESQ-R. One scale corresponds to inhibitory control and is built by averaging the Self-Control and Self-Monitoring from the AEFI and the emotional and behavioural regulation scales from the ESQ-R. The other composite score corresponds to planning and is built by averaging the Planning and Initiative sub-scale from the AEFI and three sub-scales from the ESQ-R: plan management, time management and organisation.

In our approach, tasks were aggregated to the model based on their correlation coefficient with the dependent variable, with those exhibiting higher correlations introduced first. This hierarchical procedure involved systematically adding each EF task to the model in order of their respective correlation strengths. By prioritising tasks with higher correlation coefficients, we aimed to first assess the most influential tasks in terms of their relationship with the dependent variable.

This method of aggregating tasks allowed us to evaluate the cumulative impact of the EF tasks on the overall model, starting with the most predictive and gradually including others based on their decreasing correlation coefficients. Such an approach ensured a structured and data-driven inclusion of tasks. It also allowed us to observe how the introduction of each task altered the model and to understand the incremental contribution of each task in explaining the variance in the dependent variable.

Internal consistency of the tree scales built from the AEFI and the ESQ-R were calculated with McDonald’s omega. Cronbach’s alpha was also calculated to facilitate comparisons with other studies since it is a more common measure, despite being less accurate than the omega score.

## Results

Table 1 presents descriptive statistics from the investigated variables in Study 1 and Study 2. Correlation coefficients were also calculated for both studies and are presented in Supplementary Tables S1, S2, respectively. We conducted a paired-samples t-test comparing the mean scores of free and regulated experiences from the Emotional Experience Task to determine the reported effects of regulatory strategies. The results indicated a significant and substantial effect of regulation on emotional experience [*t* (244) = 13.61, *p* < 0.001, *d* = 0.87], with the mean score for free experience being 5.68 (SD = 1.88) and for regulated experience being 4.51 (SD = 1.7).

**Table 1:** Descriptive statistics from Study 1.

	*n*	Mean	Std. deviation
Study 1			
Emotion regulation	248	1.12	1.31
Inhibitory control – Stroop	246	7.05	12.1
Switching – Dual task 1	248	17	16.43
Working memory – Letter digit	213	5.03	2.07
Study 2			
Emotion regulation	180	1.329	1.271
Inhibitory control – Stop signal	158	55.237	9.47
Switching – Dual task 2	180	5.718	6.982
Working memory – Mental rotation task	176	34.192	15.671
Inhibitory control – Questionnaires	180	8.033	1.833
Planning – Questionnaires	180	11.457	1.948
Attention – Questionnaires	180	6.767	1.442

Dual task 1, the task used for study 1; Dual task 2, the task used for study 2.

Internal consistency was calculated for the three scales used in Study 2. In the case of the inhibitory control scale, it consisted of 12 items and its internal consistency was found to be good (*ω* = 0.784, 95% confidence interval = [0.738–0.83], *ɑ* = 0.776, 95% confidence interval = [0.724–0.82]). Planning included 23 items and also presented good internal consistency (*ω* = 0.78, 95% confidence interval = [0.734–0.826], *ɑ* = 0.765, 95% confidence interval = [0.715–0.807]). However, the attention scale presented poor internal consistency (*ω* = 0.591, 95% confidence interval = [0.489–0.694], *ɑ* = 0.577, 95% confidence interval = [0.457–0.674]) and consisted of only three items.

In order to evaluate the predictive power of cognition in downregulation, several multiple regression models were tested in Study 1 and Study 2 using a hierarchical procedure. In Study 1, the first model consisting of age and sex significantly predicted downregulation [*F* (2, 246) = 7.067, *p* = 0.001, *R*2 = 0.055]. The second model included switching along sex and age, and significantly increased the regression coefficient [*F* (3, 246) = 8.792, *p* < 0.001, *R*2 = 0.098; Δ*R*2 = 0.043, *p* < 0.001], with higher switching capacity (i.e., lower scores) reflecting better ER (i.e., higher scores). The third model incorporated the experimental measure of inhibitory control [*F* (4, 244) = 6.406, *p* < 0.001, *R*2 = 0.096; Δ*R*2 = 0.003, *p* = 0.391] and the fourth model working memory as measured by the letter-digit task [*F* (5, 209) = 4.048, *p* = 0.002, *R*2 = 0.09; Δ*R*2 = 0.004, *p* = 0.346]. Neither of the latter two models significantly improved the regression coefficient. Table 2 presents details of the second model. Results from models 1 to 4 can be found in Supplementary Table S3.

**Table 2:** Summary of Model 2 from Study 1 and Study 2.

Study		*B*	Standard error	*β*	*t*	*p*
1	(Intercept)	1.149	0.300		3.834	<0.001
	Age	0.020	0.013	0.099	1.599	0.111
	Sex (male)	−0.633	0.172		−3.678	<0.001
	Switching – Dual task 1	−0.017	0.005	−0.210	−3.410	<0.001
2	(Intercept)	2.072	0.489		4.241	<0.001
	Age	−0.020	0.022	−0.069	−0.942	0.347
	Sex (male)	−0.243	0.199		−1.217	0.225
	Switching – Dual task 2	−0.038	0.013	−0.209	−2.854	0.005

B, unstandardised; β, standardised.

For study 2 a similar procedure was followed, building the first model with the demographic variables as predictors of downregulation (*F* (2, 179) = 1.33, *p* = 0.267, *R*2 = 0.015). The second model introduced switching which resulted in a statistically significant model with a significant increase in the regression coefficient [*F* (3, 179) = 3.637, *p* = 0.014, *R*2 = 0.058; Δ*R*2 = 0.044, *p* = 0.005], again, with higher switching capacity reflecting better ER. The third and fourth model included working memory as measured by the mental rotation task [*F* (4, 175) = 2.334, *p* = 0.058, *R*2 = 0.052; Δ*R*^2^ = 0, *p* = 0.983] and inhibitory control as measured by the stop-signal task [*F* (5, 153) = 1.571, *p* = 0.172, *R*^2^ = 0.050; Δ*R*^2^ = 0.001, *p* = 0.630], respectively. The final model included the scores from the three scales (i.e., attention, inhibitory control and planning). However, the model did not significantly predict downregulation and did not provide an increase in the regression coefficient [*F* (8, 153) = 1.225, *p* = 0.288, *R*^2^ = 0.063; Δ*R*^2^ = 0.013, *p* = 0.574]. Table 2 summarises model 2 and Supplementary Table S3 presents the results from models 1 to 5.

## Discussion

The current study aimed to delve into the complex interplay between EFs and emotional downregulation, encompassing two distinct but complementary research designs. Of particular interest was whether specific components of EFs—namely working memory, inhibitory control, and switching—were predictive of effective emotional downregulation capabilities. While the overarching results demonstrated a connection between EFs and emotional regulation, one aspect stood out: the ability to switch attention or multitask emerged as the sole significant predictor of emotional downregulation. This finding allows us to better understand the role of executive function in emotion regulation by isolating switching as a crucial component in the regulation of emotional states. In contrast, working memory and inhibitory control did not yield significant predictive power in this context. This nuanced outcome suggests a more targeted relationship than previously understood. Our results thereby propose a targeted avenue for further research and interventions, emphasising the role of switching or cognitive flexibility in emotional well-being.

In the first study, a focus on experimental tasks allowed us to assess the relationship between EFs (i.e., working memory, inhibitory control, and switching) with emotional downregulation. The results highlighted the predictive nature of these executive functions, especially switching, in determining one’s ability to regulate emotion effectively. These outcomes resonate well with existing literature that has observed a link between EFs and emotional control, though our study could not replicate findings linking working memory with emotion regulation (Schmeichel et al., 2008; Schmeichel and Demaree, 2010; Hendricks and Buchanan, 2016).

The second study enriched these insights by incorporating self-report measures. This approach provided a broader, ecologically valid understanding of how EFs manifest in daily emotional regulation. Interestingly, the self-report measures did not significantly predict downregulation, raising questions about the gap between self-perception and actual cognitive abilities, and the concurrent validity between experimental tasks and instrumental behaviour. It’s important to note that the attention scale displayed poor internal consistency in our study. This suggests that the measure itself may need further refinement for subsequent research in Ecuadorian population. This limitation could also account for the absence of a discernible association between attention and the variables under study.

### Sex and age in downregulation

The contrasting results concerning demographic predictors across the two studies call for more in-depth investigation. In Study 1, sex emerged as a significant predictor of emotional downregulation capabilities, with women outperforming men. However, age did not show a similar predictive value. These findings indicate the importance of careful task selection and methodological design in future research. Given that the average age for participants in both studies was 22 years, and women constituted 68% of the sample in each, future research could benefit from including a more diverse age range and a balanced gender distribution. This would allow for a more comprehensive understanding of how these demographic factors interact with emotional downregulation and executive functions.

### Inhibitory control and emotion regulation

The absence of a discernible relationship between inhibitory control and downregulation of emotion presents an intriguing question, especially given the intuitive assumption that the two constructs should be intrinsically linked. One would naturally expect that a higher degree of inhibitory control, which allows individuals to suppress or override undesired thoughts, impulses, or emotions, would correlate with more effective downregulation of emotion. However, if empirical evidence does not support this presumption, several compelling theoretical and methodological implications arise.

From a theoretical perspective, the lack of a relationship could challenge the prevailing frameworks that conceptualise emotional regulation as a form of self-control or executive functioning. If inhibitory control and emotional downregulation are not as correlated as one might expect, this could suggest that different cognitive processes or neural pathways may underlie each ability. Alternatively, it might indicate that emotional downregulation involves a broader set of psychological skills and resources, such as cognitive flexibility or emotional intelligence, that inhibitory control alone cannot capture.

From a methodological standpoint, it may raise questions about the validity of the measures used to assess either inhibitory control or emotional downregulation. If the tools employed are not sensitive enough to capture the nuances or complexities inherent in these psychological constructs, this could explain the absence of a relationship. It may also stimulate debate on whether the failure to observe a relationship is an artefact of research design or a genuine psychological phenomenon. The study by Gärtner et al. (2022) presents an important insight in this matter. In their study, a very thoroughly assessment was conducted on inhibitory control and emotion regulation. This allowed them to obtain a single score including the shared variance from six inhibitory control tasks, thus, reducing the incidence of measurement error and unique variance of each task that could be regarded as other processes involved in specific task performance. Despite this methodological precautions no significant association was found. However, only one emotion regulation strategy was investigated (i.e., detachment), which could indicate that the impact of inhibitory control on emotion regulation may follow a different path.

### Working memory and emotion regulation

The lack of a significant relationship between working memory and emotional downregulation in our study is similarly intriguing, particularly when contrasted with prior studies that have observed a connection between these two constructs (Schmeichel et al., 2008; Schmeichel and Demaree, 2010; Hendricks and Buchanan, 2016). We would expect that a well-functioning working memory would contribute positively to emotional regulation, given that it allows individuals to hold and manipulate emotional information temporarily. This capacity should, in theory, facilitate cognitive reappraisal strategies often crucial for effective emotional regulation.

This lack of association between working memory and emotional downregulation may suggest that the mechanisms underlying emotion regulation are more complex than previously thought. It could be that working memory, while important for many aspects of cognitive control, is neither directly nor broadly implicated in the specific processes that underpin emotional downregulation. This could point to a more intricate interplay of cognitive functions and emotional regulation strategies that cannot be easily captured.

The absence of an observed association between working memory and emotional downregulation in our study might also be attributable to challenges inherent in the assessment of working memory. Traditional experimental tasks used for evaluating working memory, such as the n-back task and backward digit span tasks, have been criticised for their lack of reliability (Jaeggi et al., 2010) or for primarily targeting other cognitive processes (St Clair-Thompson, 2010; St Clair-Thompson and Allen, 2013). It is plausible that the tasks we employed to assess both working memory and emotional downregulation were not sufficiently refined to detect the specific types of cognitive processing most relevant to emotion regulation. This hypothesis gains credibility when considering that we did not instruct participants to use any particular strategy for emotion regulation. Thus, considering the significant associations observed in other studies, it seems reasonable to assume that individual differences in working memory capacity may exert a more significant influence on some regulatory strategies than on others.

### Switching and emotion regulation

The consistent and significant association between switching ability and emotional downregulation observed in our study suggest that individuals with a higher degree of cognitive flexibility are better equipped to modulate their emotional responses effectively, perhaps by seamlessly switching between different emotional regulation strategies as the situation demands as described in the Cognitive Control Framework of emotion regulation flexibility presented by Pruessner et al. (2020).

One possible explanation for this association could be rooted in the neurocognitive substrates that underpin both switching and emotional regulation. Both processes are thought to engage prefrontal cortical regions known for their role in executive functions and self-regulation. The dorsolateral prefrontal cortex, for example, is implicated in both cognitive flexibility (Hampshire and Owen, 2005; Ravizza and Carter, 2008; Friedman and Robbins, 2022) and the downregulation of emotional responses (Keuper et al., 2018; Nejati et al., 2021). Thus, it is conceivable that the same neural circuits are being tapped for both abilities, providing a neurological basis for their observed association.

Another angle to consider is that emotion regulation often requires a form of mental agility. It involves the ability to disengage from an emotional response, re-appraise the situation, and then engage in a more adaptive emotional response if necessary. This series of cognitive operations bears striking resemblance to the task-switching capabilities measured in our switching tasks, where participants had to adaptively change their mental sets to respond to different task demands. In this sense, it would seem that our methods placed an emphasis on this aspect of emotion regulation, since trials requiring regulation and free experience were presented pseudorandomly.

Moreover, the capacity for effective switching could be particularly advantageous in real-world scenarios that require rapid adaptation to emotionally charged situations. Whether it’s a sudden change in a personal relationship, a stressful workplace event, or an unexpected societal upheaval, the ability to flexibly switch between different emotional regulation strategies could be a key factor in maintaining emotional well-being. Future studies could investigate more in detail whether emotion regulation flexibility (Pruessner et al., 2020) improves downregulation capacity.

### Final thoughts

One of the study’s strengths is its blend of experimental tasks and self-reported measures, which together offer a robust, multifaceted view of the EF-ER relationship. Additionally, the use of different cognitive tasks across the two studies enhances the generalisability of our findings. However, it is crucial to acknowledge the limitations. While the replication of results across two studies is a significant strength, it is important to consider that a single, more comprehensive study evaluating each EF with more than one task could offer more robust evidence. This could be achieved, for instance, by using diverse EF tasks and extracting a latent factor. Also, for both studies, we employed a hierarchical procedure to assess the contribution of each EF. In this approach, we introduced the outcome scores from the tasks demonstrating the highest correlations first. It is important to acknowledge that individual EFs, while analysed separately, often correlate with each other and may share an underlying common factor (Miyake and Friedman, 2012). Consequently, this hierarchical procedure might overestimate the contribution of the first EF introduced into the model.

Our sample predominantly comprised university students, which may limit the generalisability of our findings to a broader demographic. We also did not account for psychopathology or clinical symptoms, which could mediate the relationship between executive functions and emotion regulation. Future research would benefit from including a more diverse population to ensure the findings are widely applicable. Moreover, controlling for clinical symptoms would facilitate an assessment of their influence on this relationship. Existing literature suggests that the challenges in establishing significant associations between emotional regulation and EFs often relate to methodological decisions. In our study, the emotion regulation task we employed was particularly reliant on an individual’s ability to switch flexibly between emotional states. This specific focus could influence the nature and strength of the observed associations between EFs and emotional regulation.

In the second study we evaluated EFs using the AEFI and the ESQ-R. While these questionnaires have previously been validated as measures of EFs, the language adaptation undertaken in this study could potentially impact their validity. To address this, we conducted an analysis of internal consistency. Nonetheless, a comprehensive examination of their psychometric properties remains necessary.

In conclusion, this study provides nuanced insights into this complex relationship, with a particular focus on the role of switching or cognitive flexibility. Our findings challenge the previously held notion that all major components of EFs, such as working memory and inhibitory control, are broadly implicated in emotional downregulation. Instead, the ability to switch attention effectively emerges as a crucial skill for managing emotional states. These results not only open up targeted avenues for further research but also have important implications for the design of interventions aimed at enhancing emotional well-being. Future research should concentrate on examining how various EFs influence different facets of emotional regulation. Special attention should be given to the selection of assessment tools, as they play a critical role in shaping our understanding of this complex interplay.

## Data availability statement

The raw data supporting the conclusions of this article will be made available by the authors, without undue reservation.

## Ethics statement

The studies involving humans were approved by the Comité científico de Facultad de Ciencias Psicológicas, Universidad de Guayaquil. The studies were conducted in accordance with the local legislation and institutional requirements. The participants provided their written informed consent to participate in this study.

## Author contributions

JR: Conceptualization, Data curation, Formal analysis, Investigation, Methodology, Project administration, Software, Writing – original draft, Writing – review & editing. JL-R: Funding acquisition, Methodology, Writing – review & editing. BR: Conceptualization, Methodology, Writing – review & editing.
