# Supplementary material for: Mind over mood: exploring the executive function’s role in downregulation
Source: Front Psychol. 2024 Jan 25;15:1322055. doi: 10.3389/fpsyg.2024.1322055 (PMC10850342; doi:10.3389/fpsyg.2024.1322055)
Supplement: Supplementary file 1 [file Table_1.DOCX]

**Supplementary materials**

| **Table S1**  **Pearson's Correlations from Study 1** | | | | | | | |
| --- | --- | --- | --- | --- | --- | --- | --- |
| **Variable** | **1** |  | **2** |  | **3** |  | **4** |
| 1. Age | — |  |  |  |  |  |  |
| 2. Emotion Regulation | 0.046 |  | — |  |  |  |  |
| 3. Inhibitory control - Stroop | -0.006 |  | -0.114 |  | — |  |  |
| 4. Multitasking – Dual task 1 | 0.150 | * | -0.202 | ** | 0.265 | *** | — |
| 5. Working memory - Letter Digit | 0.200 | ** | 0.105 |  | -0.065 |  | -0.058 |
|  | | | | | | | |

Note. RT = response time, ER = error rate

| **Table S2**  **Pearson's Correlations from Study 2** | | | | | | | | | | | | | |
| --- | --- | --- | --- | --- | --- | --- | --- | --- | --- | --- | --- | --- | --- |
| **Variable** | **1** | | **2** | | **3** | | **4** | | **5** | **6** | | **7** | |
| 1. Age | — |  |  |  |  |  |  |  |  |  |  |  |  |
| 2. Emotion Regulation | -0.084 |  | — |  |  |  |  |  |  |  |  |  |  |
| 3. Working memory - Mental rotation task | 0.064 |  | -0.036 |  | — |  |  |  |  |  |  |  |  |
| 4. Inhibitory control - Stop Signal | -0.010 |  | 0.000 |  | -0.224 | ** | — |  |  |  |  |  |  |
| 5. Multitasking – Dual task 2 | 0.061 |  | -0.213 | ** | 0.245 | ** | -0.218 | ** | — |  |  |  |  |
| 6. Inhibitory control - Questionnaires | -0.103 |  | -0.029 |  | 0.016 |  | -0.079 |  | 0 | — |  |  |  |
| 7. Planning - Questionnaires | -0.172 | * | -0.030 |  | 0.016 |  | -0.186 | * | 0.054 | 0.639 | *** | — |  |
| 8. Attention - Questionnaires | -0.126 |  | -0.020 |  | -0.085 |  | -0.055 |  | 0.023 | 0.569 | *** | 0.553 | *** |
|  | | | | | | | | | | | | | |

| Table S3  Summary of Models 1 to 4 from Study 1 | | | | | | | | | | | |
| --- | --- | --- | --- | --- | --- | --- | --- | --- | --- | --- | --- |
| Model |  | B | | Standard Error | | β | | t | | p | |
| 1 | (Intercept) | 1.014 |  | 0.303 |  |  |  | 3.341 |  | < .001 |  |
|  | Age | 0.014 |  | 0.013 |  | 0.068 |  | 1.085 |  | 0.279 |  |
|  | Sex (male) | -0.648 |  | 0.176 |  |  |  | -3.686 |  | < .001 |  |
| 2 | (Intercept) | 1.149 |  | 0.300 |  |  |  | 3.834 |  | < .001 |  |
|  | Age | 0.020 |  | 0.013 |  | 0.099 |  | 1.599 |  | 0.111 |  |
|  | Sex (male) | -0.633 |  | 0.172 |  |  |  | -3.678 |  | < .001 |  |
|  | Multitasking – Dual task 1 | -0.017 |  | 0.005 |  | -0.210 |  | -3.410 |  | < .001 |  |
| 3 | (Intercept) | 1.121 |  | 0.330 |  |  |  | 3.401 |  | < .001 |  |
|  | Age | 0.024 |  | 0.014 |  | 0.117 |  | 1.707 |  | 0.089 |  |
|  | Sex (male) | -0.594 |  | 0.192 |  |  |  | -3.098 |  | 0.002 |  |
|  | Multitasking – Dual task 1 | -0.015 |  | 0.006 |  | -0.178 |  | -2.492 |  | 0.013 |  |
|  | Inhibitory control - Stroop | -0.006 |  | 0.008 |  | -0.054 |  | -0.765 |  | 0.445 |  |
| 4 | (Intercept) | 0.969 |  | 0.367 |  |  |  | 2.639 |  | 0.009 |  |
|  | Age | 0.021 |  | 0.014 |  | 0.103 |  | 1.470 |  | 0.143 |  |
|  | Sex (male) | -0.593 |  | 0.192 |  |  |  | -3.090 |  | 0.002 |  |
|  | Multitasking – Dual task 1 | -0.014 |  | 0.006 |  | -0.172 |  | -2.410 |  | 0.017 |  |
|  | Inhibitory control - Stroop | -0.006 |  | 0.008 |  | -0.051 |  | -0.725 |  | 0.469 |  |
|  | Working memory - Letter Digit | 0.041 |  | 0.044 |  | 0.065 |  | 0.946 |  | 0.346 |  |
|  | | | | | | | | | | | |
| B = unstandardised, β = standardised | | | | | | | | | | | |

| Table S4  Summary of Models 1 to 5 from Study 2 | | | | | | | | | | | | |
| --- | --- | --- | --- | --- | --- | --- | --- | --- | --- | --- | --- | --- |
| Model | |  | B | | Standard Error | | β | | t | | p | |
| 1 |  | (Intercept) | 1.936 |  | 0.496 |  |  |  | 3.903 |  | < .001 |  |
|  |  | Age | -0.024 |  | 0.022 |  | -0.082 |  | -1.096 |  | 0.275 |  |
|  |  | Sex (male) | -0.239 |  | 0.203 |  |  |  | -1.174 |  | 0.242 |  |
| 2 |  | (Intercept) | 2.072 |  | 0.489 |  |  |  | 4.241 |  | < .001 |  |
|  |  | Age | -0.020 |  | 0.022 |  | -0.069 |  | -0.942 |  | 0.347 |  |
|  |  | Sex (male) | -0.243 |  | 0.199 |  |  |  | -1.217 |  | 0.225 |  |
|  |  | Multitasking – Dual task 2 | -0.038 |  | 0.013 |  | -0.209 |  | -2.854 |  | 0.005 |  |
| 3 |  | (Intercept) | 2.091 |  | 0.553 |  |  |  | 3.780 |  | < .001 |  |
|  |  | Age | -0.021 |  | 0.023 |  | -0.069 |  | -0.928 |  | 0.355 |  |
|  |  | Sex (male) | -0.037 |  | 0.014 |  | -0.201 |  | -2.613 |  | 0.010 |  |
|  |  | Multitasking – Dual task 2 | -0.213 |  | 0.214 |  |  |  | -0.998 |  | 0.319 |  |
|  |  | Working memory - Mental rotation task | 0.000 |  | 0.006 |  | -0.002 |  | -0.021 |  | 0.983 |  |
| 4 |  | (Intercept) | 2.165 |  | 0.600 |  |  |  | 3.610 |  | < .001 |  |
|  |  | Age | -0.027 |  | 0.024 |  | -0.093 |  | -1.142 |  | 0.255 |  |
|  |  | Sex (male) | -0.039 |  | 0.217 |  |  |  | -0.181 |  | 0.856 |  |
|  |  | Multitasking – Dual task 2 | -0.035 |  | 0.014 |  | -0.206 |  | -2.445 |  | 0.016 |  |
|  |  | Working memory - Mental rotation task | 0.002 |  | 0.007 |  | 0.020 |  | 0.229 |  | 0.819 |  |
|  |  | Inhibitory control - Stop Signal | 0.000 |  | 0.002 |  | -0.041 |  | -0.483 |  | 0.630 |  |
| 5 |  | (Intercept) | 3.098 |  | 0.942 |  |  |  | 3.289 |  | 0.001 |  |
|  |  | Age | -0.031 |  | 0.024 |  | -0.104 |  | -1.265 |  | 0.208 |  |
|  |  | Sex (male) | -0.035 |  | 0.218 |  |  |  | -0.161 |  | 0.873 |  |
|  |  | Multitasking – Dual task 2 | -0.034 |  | 0.014 |  | -0.200 |  | -2.356 |  | 0.020 |  |
|  |  | Working memory - Mental rotation task | 0.000 |  | 0.007 |  | 0.011 |  | 0.120 |  | 0.905 |  |
|  |  | Inhibitory control - Stop Signal | -0.001 |  | 0.002 |  | -0.054 |  | -0.628 |  | 0.531 |  |
|  |  | Inhibitory control - Questionnaires | -0.018 |  | 0.076 |  | -0.027 |  | -0.232 |  | 0.817 |  |
|  |  | Planning - Questionnaires | -0.018 |  | 0.069 |  | -0.029 |  | -0.264 |  | 0.793 |  |
|  |  | Attention - Questionnaires | -0.067 |  | 0.092 |  | -0.077 |  | -0.727 |  | 0.469 |  |
|  | | | | | | | | | | | | |
| B = unstandardised, β = standardised | | | | | | | | | | | | |

**Table S5**

**List of pictures used from the IAPS**

| IAPS3102 | IAPS7020 |
| --- | --- |
| IAPS3120 | IAPS3130 |
| IAPS7484 | IAPS6540 |
| IAPS7045 | IAPS1050 |
| IAPS3063 | IAPS7003 |
| IAPS3071 | IAPS3005_1 |
| IAPS8492 | IAPS2214 |
| IAPS9570 | IAPS6313 |
| IAPS3015 | IAPS2811 |
| IAPS8030 | IAPS9410 |
| IAPS1645 | IAPS7185 |
| IAPS3053 | IAPS9187 |
| IAPS3000 | IAPS6230 |
| IAPS3010 | IAPS3064 |
| IAPS7004 | IAPS2749 |
| IAPS3080 | IAPS7255 |
| IAPS3168 | IAPS4800 |
| IAPS6550 | IAPS2411 |
| IAPS8179 | IAPS3110 |
| IAPS7235 | IAPS7640 |
| IAPS3001 | IAPS2095 |
| IAPS9075 | IAPS5621 |
| IAPS3068 | IAPS2397 |
| IAPS8160 | IAPS4290 |
| IAPS2220 | IAPS3500 |
| IAPS6350 | IAPS2484 |
| IAPS4668 | IAPS3400 |
| IAPS9413 | IAPS4220 |
| IAPS7476 | IAPS4670 |
| IAPS6231 | IAPS3100 |
| IAPS3266 | IAPS8232 |
| IAPS8186 | IAPS3069 |
| IAPS9070 | IAPS3131 |
| IAPS1120 | IAPS7179 |
| IAPS3060 | IAPS4659 |
